# Sodium Valproate Combined With Topiramate vs. Sodium Valproate Alone for Refractory Epilepsy: A Systematic Review and Meta-Analysis

**DOI:** 10.3389/fneur.2021.794856

**Published:** 2022-01-05

**Authors:** Zhen-Ye Ji, Yi-Qian Huang, Wen-Zhen He

**Affiliations:** ^1^The Second Department of Neurology, First Affiliated Hospital of Shantou University Medical College, Shantou, China; ^2^Department of Internal Neurology, First Affiliated Hospital of Shantou University Medical College, Shantou, China

**Keywords:** topiramate (Topamax), sodium valproate, epilepsy, refractory epilepsy, antiepileptic drugs (AEDs)

## Abstract

**Background:** Among antiepileptic drugs (AEDs), sodium valproate alone or in the combination of topiramate (TPM) for treating refractory epilepsy was controversial. This meta-analysis aimed to systematically evaluate the clinical effects of these two regimens in this population.

**Methods:** Relevant studies up to August 2021 were identified through systematic searches of CNKI, Wanfang, PubMed, and Embase databases. We assessed the effectiveness and the frequency of absence seizures, atonic seizures, and tonic–clonic seizures. The included literature's risk of bias was evaluated using the Cochrane Collaboration's Risk of Bias tool. Sensitivity analysis was conducted to confirm the results' stability. STATA 15.0 was utilized for all pooled analyses in the included studies.

**Results:** Totally 10 articles were determined for our meta-analysis, involving 976 patients with epilepsy in total (combined group, *n* = 488; monotherapy group, *n* = 488). The results of this meta-analysis indicated that the total effective rate of sodium valproate combined with TPM was higher than that of sodium valproate alone (random-effect model: *OR* = 3.52; 95% CI 1.47 to 8.47; *p* < 0.001; *I*^2^ = 73.8%). The frequency of absence seizures in the combined group was lower (fixed-effect model: WMD = −6.02; 95% CI −6.50 to −5.54; *I*^2^ = 0.0%) than that in the monotherapy group, with a statistical difference (*p* < 0.05). The combined group had lower frequency of atonic seizures (WMD = −4.56, 95% CI −6.02 to −3.10; *I*^2^ = 82.6%) and lower frequency of tonic–clonic seizures (WMD = −3.32; 95% CI −4.75 to −1.89; *I*^2^ = 96.4%). In addition, the distinct difference of adverse events was non-existent between two groups.

**Conclusions:** Sodium valproate combined with TPM was more effective than sodium valproate alone for epilepsy therapy. This meta-analysis provides feasibility data for a larger-scale study on AED therapy of refractory epilepsy and may contribute to better therapy strategies for epilepsy clinically.

## Introduction

Epilepsy is a chronic brain disorder characterized by long-term prone epileptic seizures (1, 2). It is a complex disease with multiple risk factors and a strong genetic tendency rather than a single expression and etiology, which affects over 70 million people globally and ~80% of cases occur in developing countries (1). The number of available antiepileptic drugs (AEDs) designed to inhibit seizure occurrence has increased substantially over the decades (3). However, about a third of patients is still hindered by drug resistance (4), which is regarded as “drug-resistant” or “refractory” (5). In addition, the risk of death, psychiatric, and also adverse effects from AEDs increased remarkably in 20–30% of patients with refractory epilepsy (6). Therefore, it is very crucial to find a new effective therapy strategy of AEDs clinically.

Pharmacotherapy paradigms in epilepsy are constantly evolving. The monotherapy or multitherapy for AEDs has been a controversial topic over the last few decades. Monotherapy with AEDs is the primary initial cure for epilepsy (7). Globally, sodium valproate is one of the most generally used AEDs (8) for monotherapy treatment, which is usually regarded as a routine drug choice in adults and children with intractable epilepsy due to its broad-spectrum mechanism of action and antiepileptic activity (9). However, one study indicated that drug-resistant epilepsy patients have seizures that cannot be controlled by a single drug and requires a combination of two agents (8, 10). Therefore, AED combination therapy is becoming popular once more, with up to 30–40% of children using this treatment strategy. Ferrendelli pointed out that the era of “rational multitherapy” had begun (11). When the initial monotherapy fails, drug-resistant epilepsy almost always requires multidrug therapy, but the issue of the best cure is still debatable (12).

Topiramate (TPM) is a promising new AED as monotherapy or adjunctive therapy for generalized tonic–clonic seizures or partial seizures in adults and children (13, 14). This new AED has few distinct interactions with other drugs in clinic, which is effective when utilized in combination with other AEDs. Furthermore, oral TPM is quickly absorbed by epilepsy patients, with about 80% relative bioavailability (14). Studies have shown that TPM has the advantages of a five-fold mechanism of action, high seizure-free rate and effective rate in combination therapy, less drug interaction, and good tolerance. Therefore, it is recommended for combination therapy by domestic and foreign guidelines (15–19). Zhang reported that sodium valproate and TPM can be quickly absorbed, in which the combination therapy of the two drugs has certain advantages (20).

However, there may be safety issues during combination therapy, such as unwanted drug interactions. For example, Cheung et al. described a case report that valproate combined with TPM-induced hyperammonemic encephalopathy syndrome for a 15-year-old boy (21). Moreover, the efficacy and safety of the two therapeutic strategies are rarely evaluated in these studies. Here, we conducted a meta-analysis to estimate the clinical efficacy of sodium valproate combined with TPM vs. sodium valproate alone in refractory epilepsy therapy and to provide more guidance for the treatment of AEDs in the future.

## Methods

### Search Strategy

For this meta-analysis, two researchers independently, comprehensively, and systematically searched the literature in CNKI, Wanfang, PubMed,and also Embase databases. The literature search was limited to full-length articles published up to August 2021 in Chinese and in English. The keywords used in the search were “Topiramate OR Topamax OR TPM” and “Sodium Valproate” and “epilepsy” and “Refractory epilepsy OR Intractable epilepsy.” Two reviewers independently evaluated the qualified articles, and divergence was resolved *via* discussion, and if necessary, arbitrated by the third reviewer. Two authors independently assessed the searched studies based on the selection criteria, manually checking the retrieved articles' reference lists to determine additional relevant studies. Differences were resolved through discussion until the consensus was reached.

### Selection Criteria

Studies included in our meta-analysis were required to satisfy the following criteria: (1) be related to AED therapy for refractory epilepsy (no restriction to the type of refractory epilepsy); (2) the intervention of the experimental group was sodium valproate combined with TPM, and the intervention of the control group was sodium valproate; (3) the main outcomes in the study included effectiveness, absence seizures, atonic seizures, tonic–clonic seizures, and adverse events.

Studies were excluded from this analysis if they were (1) repeated articles; (2) summary of the meeting, comments, letters, existing systematic reviews, and meta-analysis; (3) study on TPM combined with other drugs.

### Data Extraction and Quality Assessment

The information from all qualified articles that met the inclusion criteria was extracted by two reviewers independently. Meanwhile, the risk of bias of the selected studies was evaluated to ensure the data's reliability. Any difference in the extracted data was resolved through discussion. If a disagreement still existed after the discussion, a third investigator was invited to evaluate these articles. The data extracted by the two researchers independently reading the full text of each eligible article, including the following information: the author's name, publication date, sample size, age, intervention measures, and also outcome measures.

The included studies' risk of bias was assessed using the Cochrane Collaboration's Risk of Bias tool, categorizing the study as “low risk” of bias, “high risk” of bias, or “unclear risk” of bias (32). The quality assessment was conducted by two reviewers independently; in case of disagreements, the third reviewer was consulted.

### Outcome Measures

We assessed the rate of effectiveness and the frequency of absence seizures, atonic seizures, tonic–clonic seizures, and also adverse events. The main outcome for measuring efficacy was the total effective rate. The total effectiveness was defined as the improvement of symptoms and signs, mainly including sudden, recurrent loss of consciousness, body convulsing, and frothing at the mouth, and also a decrease of ≥50% in seizure frequency from baseline to posttreatment. The other outcome was the condition of patients who suffered from treatment-emergent adverse events (TEAEs). TEAE is an adverse event that occurred or became worse in the treatment phase (33). TEAEs evaluated in our meta-analysis included headache, loss of appetite, and nausea.

### Statistical Analysis

All analyses were conducted using STATA 15.0 software. The combined effect was assessed adopting weighted mean difference (WMD) and 95% CI or odds ratios (OR) value and 95% CI. The frequency of seizures was described by mean and standard deviation, and the pooled effect analysis in this meta-analysis was performed on the mean of seizures in each study. Statistical methods for combining the results of studies generally weight the influence of each study by the inverse of the variance for the estimated measure of effect. Based on the heterogeneity test results, the total effects were evaluated through random-effect or fixed-effect models (34, 35). *Q*-test and *I*^2^-test were applied to assess the heterogeneity between studies. The fixed-effect model was used in case of *p* > 0.1, *I*^2^ ≤ 50%; Otherwise, the random-effect model was used. Sensitivity analysis was used for estimating the results' stability. A funnel plot combined with Egger's test was used to assess publication bias (36, 37). The *p-*value < 0.05 indicates that the difference was statistically significant.

## Results

### Literature Search and Study Characteristics

**Figure 1:**
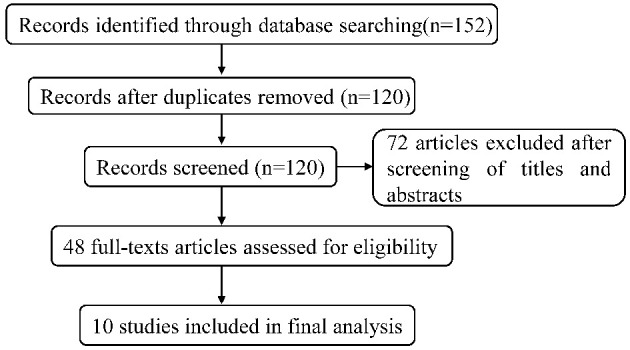
The retrieval flow chart for the meta-analysis.

Overall, 152 articles were searched by the systematic search in total, of which 32 duplicates were excluded. After independently screening the title and abstract by two review authors, 72 irrelevant articles were removed, and 48 studies were selected for detailed full-text review. In the light of selection criteria, 38 articles were eliminated, and 10 were eventually included in our meta-analysis. The flow chart (Figure 1) described the process of literature retrieval and selection. The basic information of the included studies was summarized in Table 1. We identified a total of 976 patients with refractory epilepsy included in 10 reports of controlled studies (22–31) (Table 1). The cases of each study were selected and divided into a control group and an observation group. Sodium valproate was given to the control group, while the observation group was with the combination of TPM. The treatment effects were compared between two groups. All studies were randomized and nine studies reported the effective rate in groups of combined treatment vs. control groups (Table 1).

**Table 1:** The baseline characteristics of the included studies.

**References**	**Group**	**Intervention**	**Sample**	**Age**	**Outcomes**
Chen et al. (22)	T + S	T: 300 mg, once/day; S: 15 mg/kg, once/day, 12 weeks	30	48.24 ± 1.27	(1), (5)
	S	S: 15 mg/kg, once/day, 12 weeks	30	48.34 ± 1.32	
Wang et al. (23)	T + S	T: adult, 25 mg, child, 0.5 mg, two times/day, 3 months; S: adult, 1 g, child, 30 mg, two times/day, 3 months	33	27.32 ± 13.81	(1), (2), (3), (4)
	S	S: adult, 1 g/day, child, 30 mg/day, 3 months	33	26.71 ± 14.55	
Qu (24)	T + S	T: 400 mg/day; S: 5–10 mg/kg/day, 1 week; after 1 week, 10–20 mg/kg/day	80	31.3 ± 5.7	(2), (3), (4)
	S	S: 5–10 mg/kg/day, 1 week; after 1 week, 10-20 mg/kg/day	80	31.2 ± 5.5	
Wlr et al. (25)	T + S	T: 100–150 mg/day; S: 0.2 g, once/day, 2 weeks; after 2 weeks, two times/day, maximum ≤ 1.2 g, 6 months	53	32.11 ± 2.36	(1), (2), (3), (4), (5)
	S	S: 0.2 g, once/day, 2 weeks; after 2 weeks, two times/day, maximum ≤ 1.2 g, 6 months	53	32.10 ± 2.35	
Li et al. (26)	T + S	T: 25–50 mg/day; S: 15 mg/kg, three times/day	50	45 ± 2.3	(1), (5)
	S	S: 15 mg/kg, three times/day	50	46 ± 2.1	
Yan and Dai (27)	T + S	T: 100–150 mg/day; S: 0.2 g, once/day, 2 weeks; after 2 weeks, two times/day, maximum ≤ 1.2 g	62	37.6 ± 4.2	(1)
	S	S: 0.2 g, once/day, 2 weeks; after 2 weeks, two times/day, maximum ≤ 1.2 g	62	37.4 ± 4.3	
Peng et al. (28)	T + S	T: 100–150 mg, two times/day; S: 0.2 g, once/day, 2 weeks; after 2 weeks, two times/day, maximum ≤ 1.2 g	40	40.28 ± 7.36	(1)
	S	S: 0.2 g, once/day, 2 weeks; after 2 weeks, two times/day, maximum ≤ 1.2 g	40	40.59 ± 7.14	
Chen and Pan (29)	T + S	T: 300 mg/day; S: 15 mg/kg, once/day, 12 weeks	75	47.9 ± 6.8	(1), (5)
	S	S: 15 mg/kg, once/day, 12 weeks	75	48.7 ± 6.9	
Liu (30)	T + S	T: adult, 25 mg, child, 0.5 mg, two times/day; S: adult, 500 mg, child, 15 mg, two times/day	15	36.7 ± 11.2	(1)
	S	S: adult, 500 mg, child, 15 mg, two times/day	15	37.7 ± 10.9	
Wang et al. (31)	T + S	T: 300 mg/day; S: 5–10 mg/kg, two times or three times/day, 12 weeks	50	–	(1), (5)
	S	S: 5–10 mg/kg, two times or three times/day, 12 weeks	50	–	

*T, Topamax; S, sodium valproate; (1) effective rate; (2) absence seizure; (3) atonic seizures; (4) tonic–clonic seizure; (5) adverse events (headache, loss of appetite, and nausea)*.

### Quality Assessment of Included Studies

**Figure 2:**
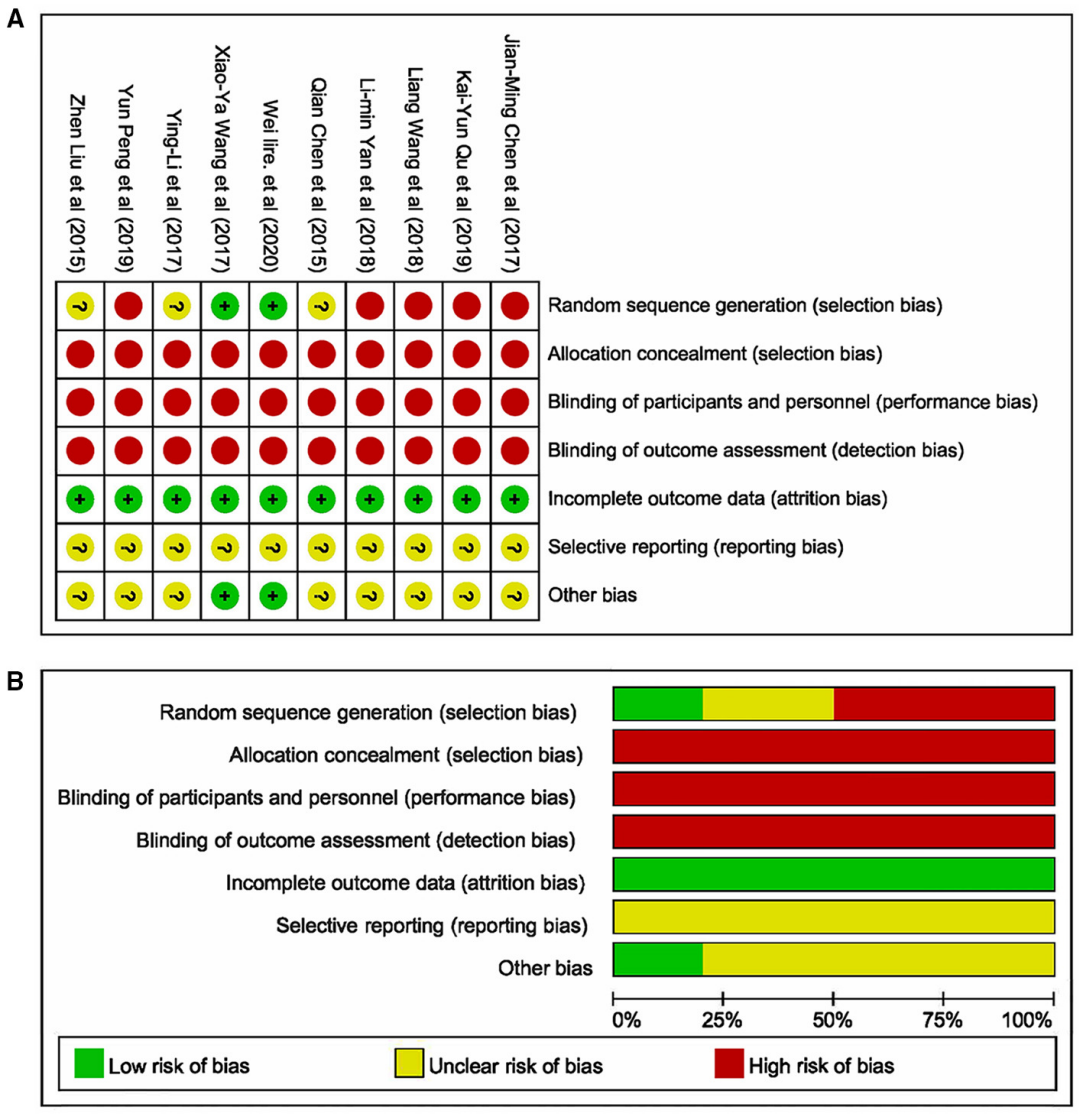
Methodological quality of studies included in this meta-analysis. **(A)** Summary of the risk of bias in the included studies; **(B)** Risk of bias graph: the articles' risk of bias evaluation results.

The literature's risk bias estimation results were presented in Figure 2. As displayed in Figure 2A, two articles described the method of randomization. All studies did not describe stratified seclusion, and the corresponding risk was judged as the high risk; none of the included studies were blinded, which was a high risk. All research data were completed and without missing (Figure 2B). The included studies' selection bias was unknown, and other sources of biases were unknown.

### Efficacy

**Figure 3:**
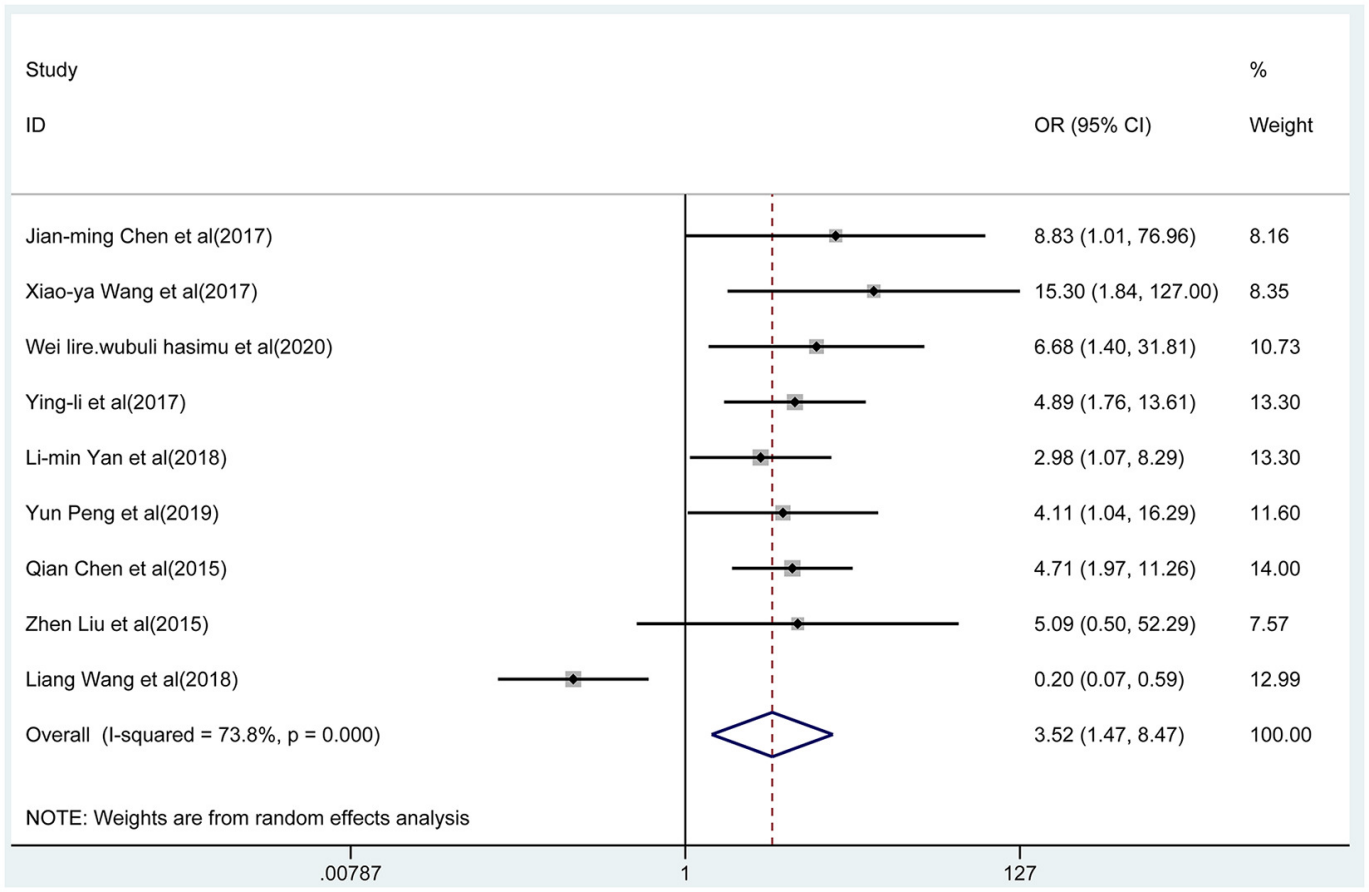
Forest plot of the overall effectiveness of the combined group vs. monotherapy group.

**Figure 4:**
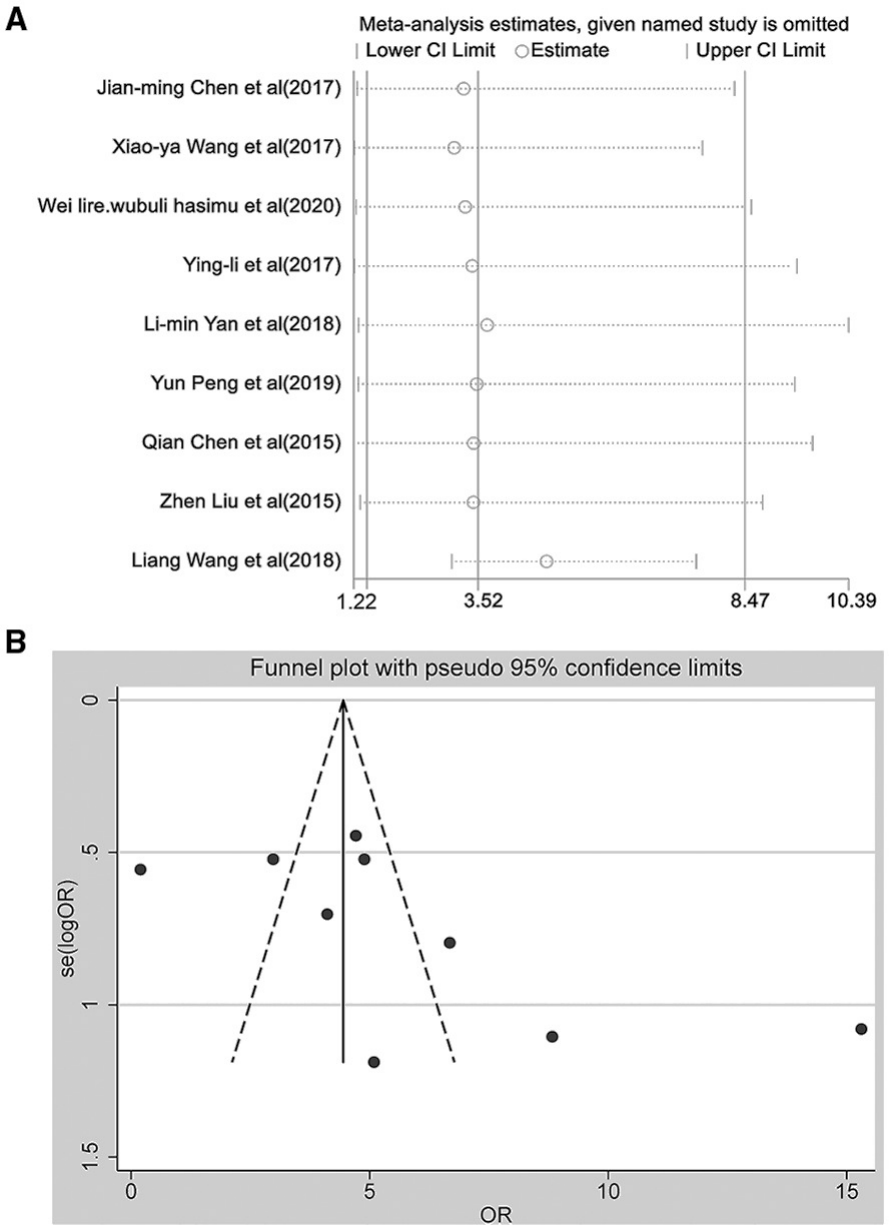
Sensitivity analysis of results **(A)** and funnel plot of the publication bias **(B)**.

Nine articles reported the total effective rate of the combined group and monotherapy group (Figure 3). The total effective rate of sodium valproate combined with TPM was higher than that of sodium valproate alone (random-effect model: *OR* = 2.17, 95% CI 1.47-8.47), and the difference was statistically significant (*p* < 0.05). By removing each qualified study in turn, we conducted the sensitivity analysis to evaluate the impact of each individual study on the pooled OR (Figure 4A). The estimated value of the total effect was within the range of 95% CI (1.47–8.47), which suggested the results were stable and reliable in this meta-analysis. The funnel plot was visually observed to estimate the publication bias, and no obvious asymmetry was found (Figure 4B). Moreover, the Egger's test was used for quantifying the publication bias, and the *p*-value was 0.09, indicating that the studies' biases were non-existent.

### Absence Seizures, Atonic Seizures, and Tonic–Clonic Seizures

**Figure 5:**
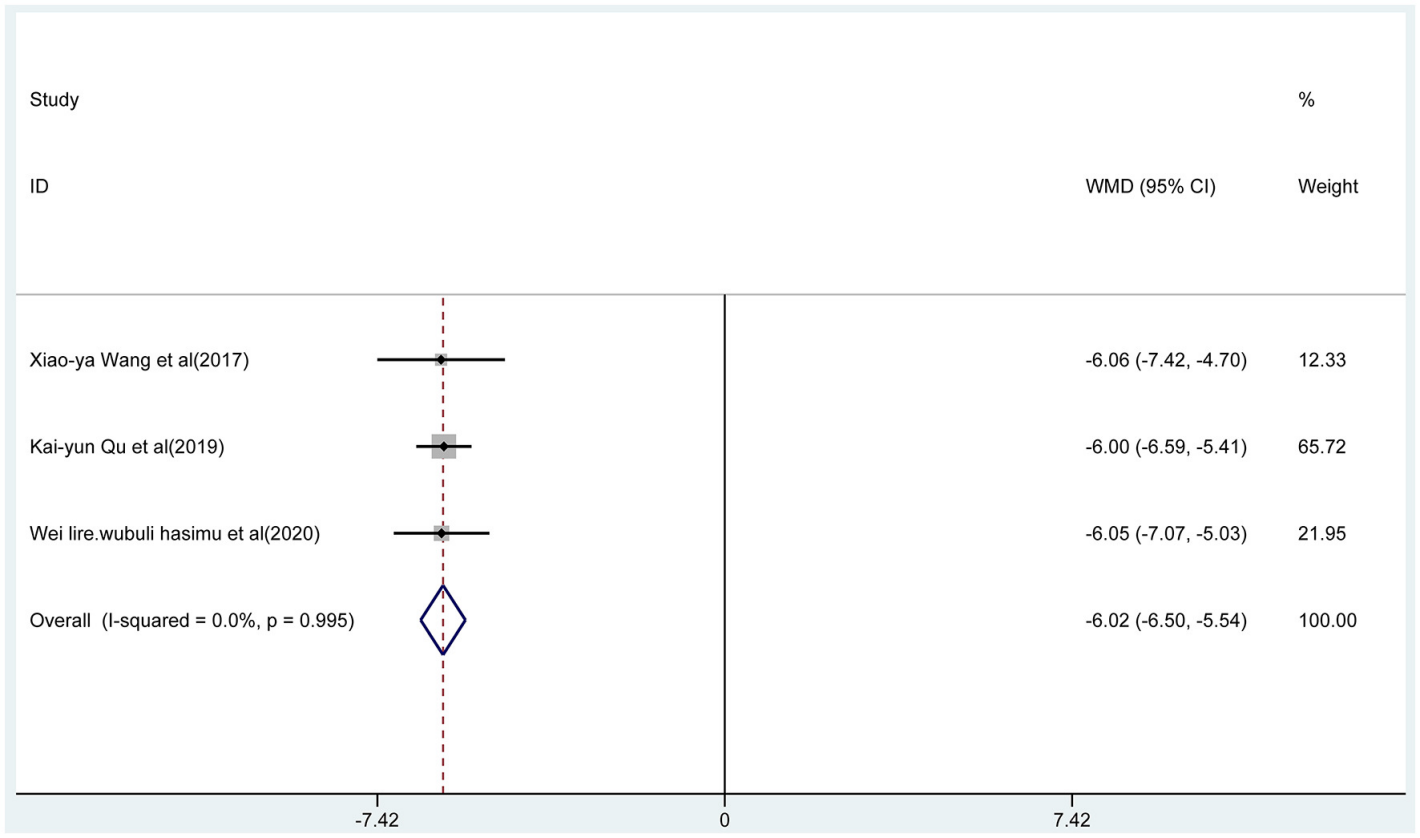
The forest plot of absence seizures, the combined group vs. monotherapy group.

**Figure 6:**
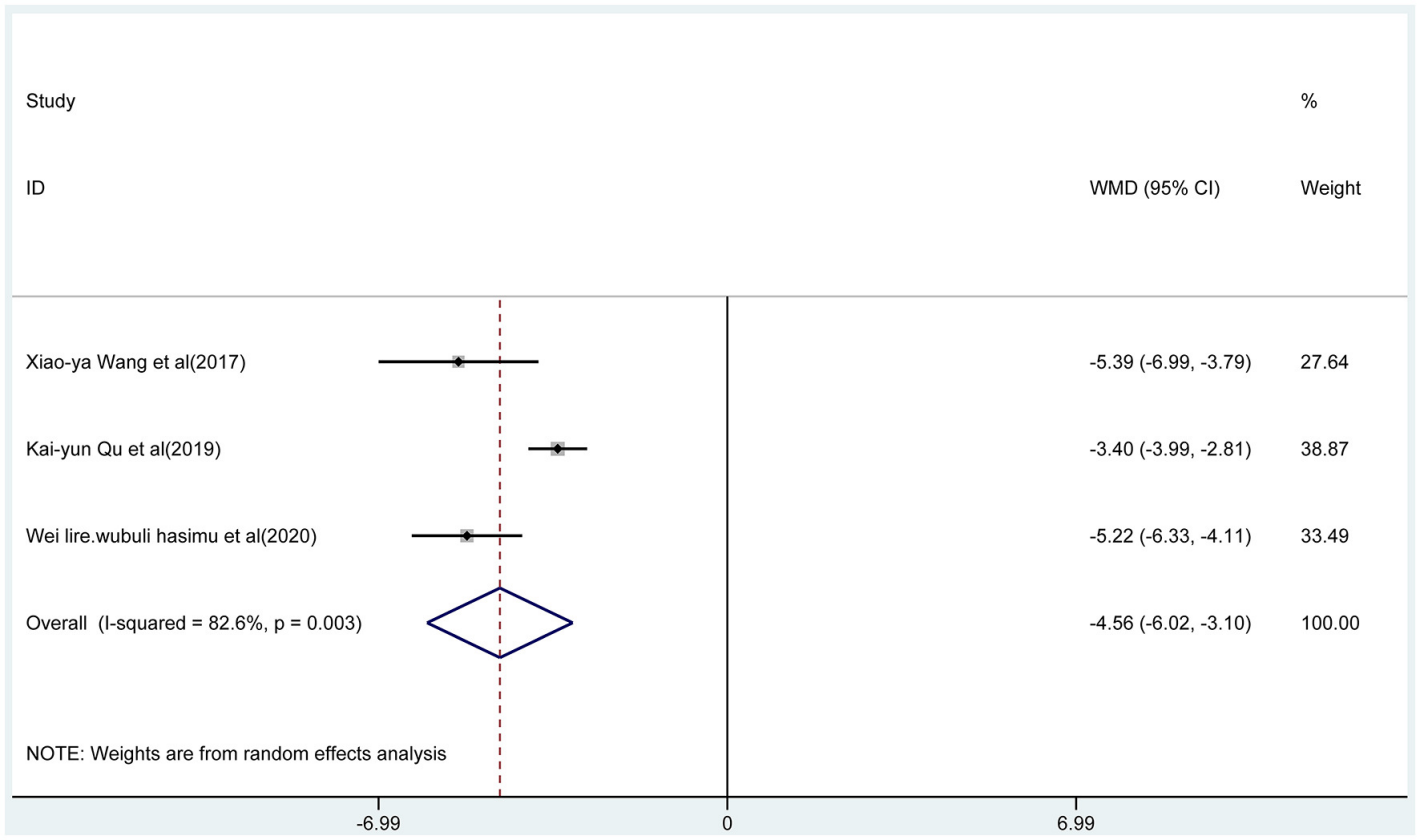
The forest plot of atonic seizures, the combined group vs. monotherapy group.

**Figure 7:**
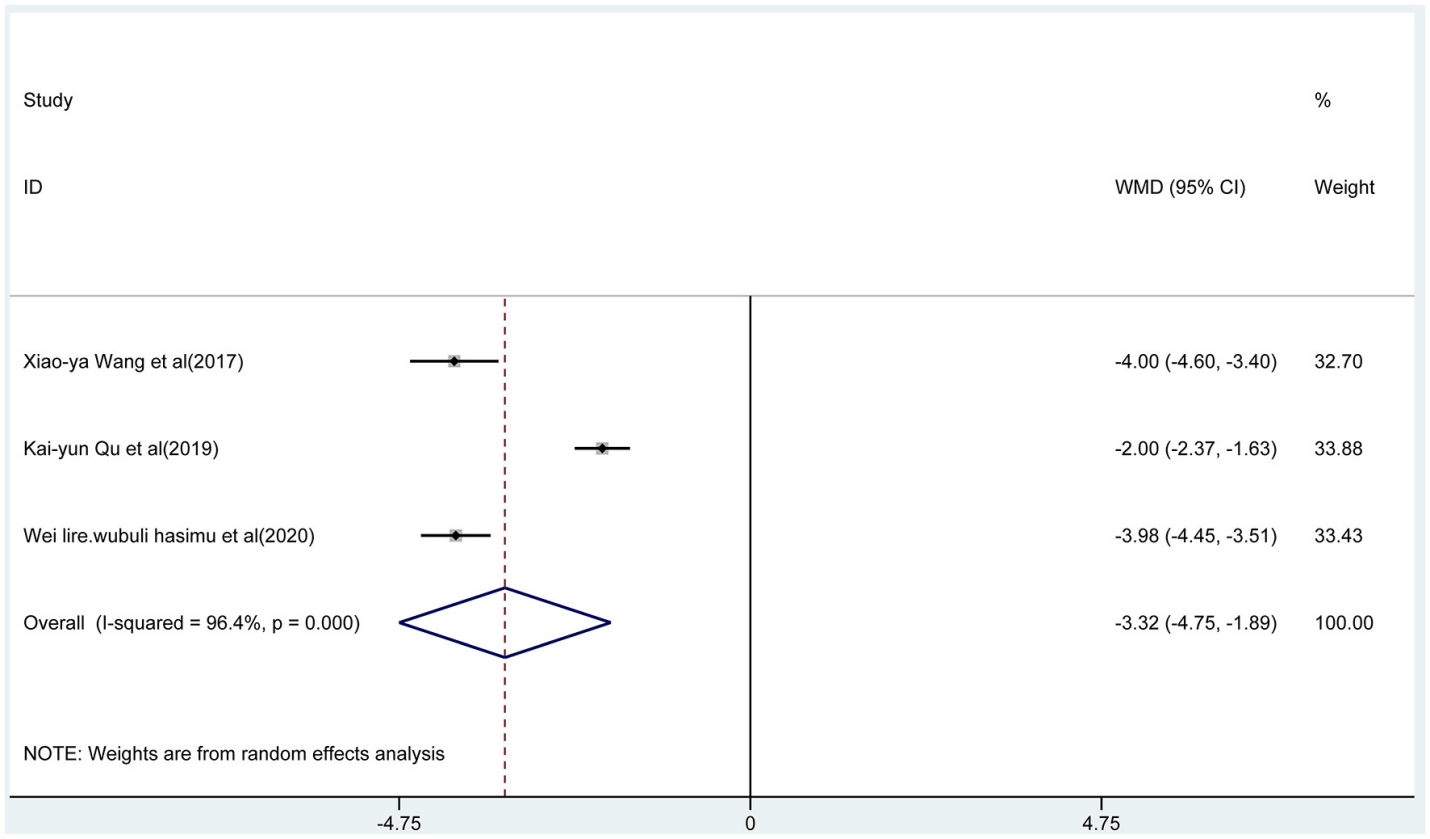
The forest plot of tonic–clonic seizures, the combined group vs. monotherapy group.

Three articles reported the frequency of absence seizures, atonic seizures, and tonic–clonic seizures, respectively. The results showed that the frequency of absence seizures of sodium valproate combined with TPM was lower (fixed-effect model: WMD = −6.02; 95% CI −6.50 to −5.54; *I*^2^ = 0.0%) than that of sodium valproate alone, with statistically significant difference (*p* < 0.05; Figure 5). Likewise, according to the random-effect model, the results showed that the frequency of atonic seizures (WMD = −4.56; 95% CI −6.02 to −3.10; *I*^2^ = 82.6%; Figure 6) and tonic–clonic seizures (WMD = −3.32; 95% CI −4.75 to −1.89; *I*^2^ = 96.4%; Figure 7) in the combined group was less than that in monotherapy group.

### Adverse Events

**Figure 8:**
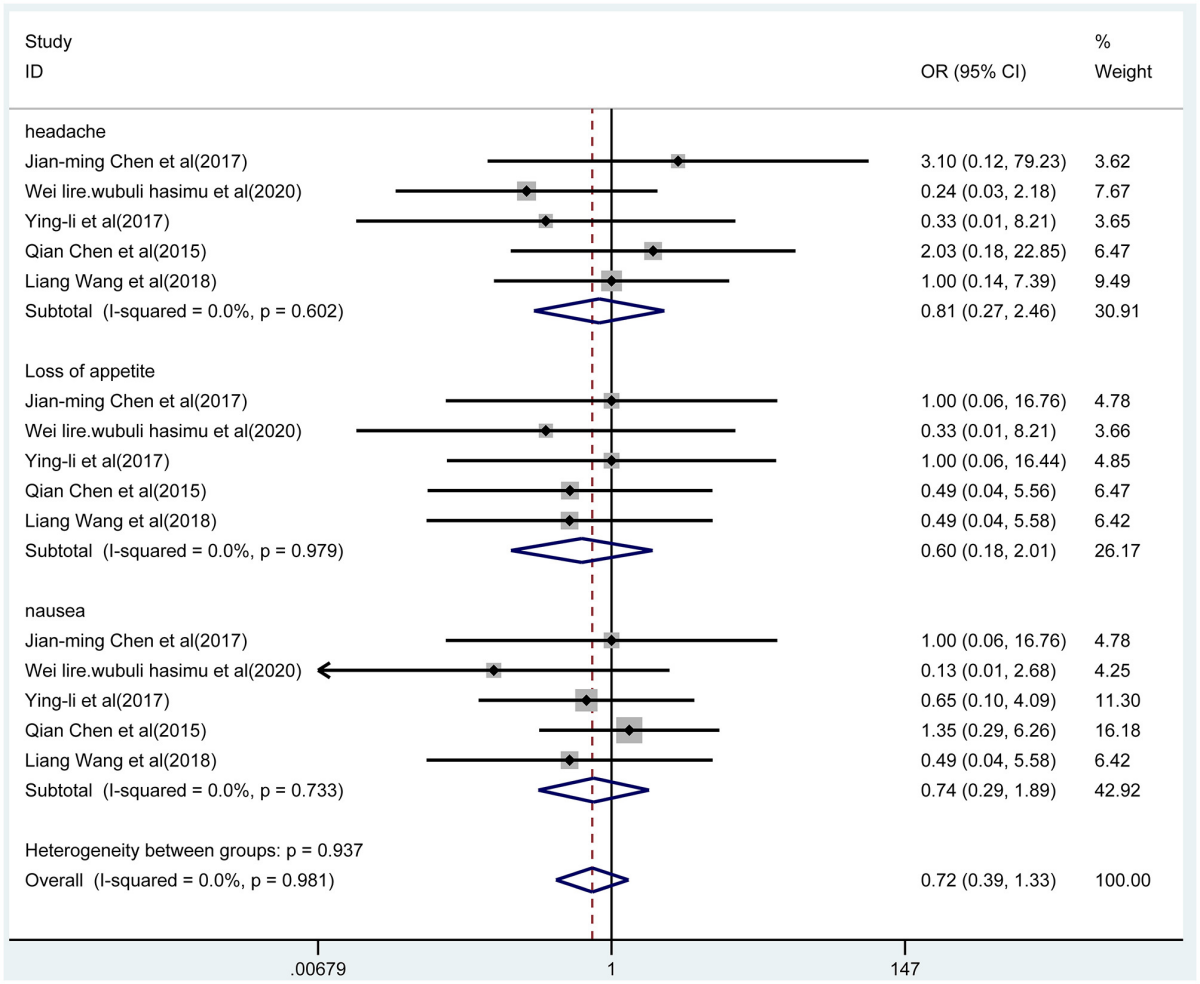
The forest plot of adverse events, the combined group vs. monotherapy group.

We pooled data on adverse events mainly including headache, loss of appetite, and nausea. Five studies reported these adverse events with the two treatments, which were presented in Figure 8. According to the fixed-effect model, the results indicated that there was no significant difference in adverse events between the combined group and the monotherapy group (*OR* = 0.72, 95% CI 0.39-1.33; *p* = 0.297; *I*^2^ = 0.0%, *p* = 0.981). In addition, the results of subgroup analysis indicated that there were no differences in the incidence of headache (*p* = 0.710), anorexia (*p* = 0.410), and nausea (*p* = 0.527) between the two groups.

## Discussion

Epilepsy is one of the most common neurological disorders, leading to a considerable incidence rate (38). In clinical practice, drugs are often used to control the condition of patients, thereby reducing the incidence of patients with intractable epilepsy and improving the quality of life and treatment effect (22). However, the patients with chronic intractable “drug-resistant” epilepsy cannot obtain the freedom of continuous seizures after a trial of two AEDs, thus requiring treatment with a combination of drugs (39). Herein, our meta-analysis indicated that sodium valproate in combination with TPM contributed to better control of seizures, which may be a good option for refractory epilepsy therapy. The choice of initial pharmacotherapy for epilepsy should be mainly guided by the evidence of efficacy and safety, so as to ensure that the ultimate goal is to keep seizure free without intolerable adverse events (40).

To date, many researchers have compared the efficacy of sodium valproate combined with TPM vs. sodium valproate alone for refractory epilepsy. Nevertheless, there is still a lack of systematic collection, classification, and assessment of these research data. In our study, 10 articles were screened strictly based on the selection criteria, and 976 patients with refractory epilepsy were included, with the age range of 10-70 years. The results fully indicated that sodium valproate combined with TPM has significant curative effect on refractory epilepsy compared with sodium valproate alone, which can reduce epilepsy attack frequency. Here, we conducted the meta-analysis to provide the comprehensive and explicit evidence-based evaluation of relative efficacy of the two therapeutic strategies. To our knowledge, this meta-analysis was the first evaluation in regard to clinical effect of sodium valproate combined with TPM vs. sodium valproate alone for refractory epilepsy.

There were similar design features among included studies, and the low heterogeneity between trials enhances the estimates' accuracy (41). The results fully indicated that total effective rate of the combined group was significantly higher than that of the monotherapy group. Notably, the frequency of absence seizures, atonic seizures, and tonic–clonic seizures decreased significantly in the combined group. In addition, compared with monotherapy, the adverse events of patients during the combination therapy were not increased. Overall, all evaluation results of this meta-analysis fully indicated that the efficacy of sodium valproate combined with TPM was better than that of sodium valproate alone.

Sodium valproate, as a typical broad-spectrum AED, has become the first choice for treating epileptic absence seizures and generalized tonic–clonic seizures in clinical practice (8). It has the pharmacological effect involving a variety of mechanisms, including blockade of voltage-gated sodium channels, reduced effect of excitatory amino acids, and also potentiation of γ-aminobutyric acid (GABA) ergic transmission (42). TPM, a structurally novel broad-spectrum AED, also has the same mechanism of action as sodium valproate, including the state-dependent inhibition of sodium channels, the potentiation of GABA-induced chloride influx, and the blockade of glutamate-related excitatory neurotransmission (43). In this meta-analysis, we found that the efficacy of sodium valproate in combination with TPM was obviously superior to sodium valproate alone, which was identical with the literature reported (44). We speculate that abnormal neuronal excitability associated with seizures suppressed through the two drugs' common mechanism of block the persistent Na^+^ current can account for decline of seizure frequency. Moreover, one study reported that TPM-induced reduction in tonic–clonic seizures corresponded well with the decrease of glutamate levels in SER (45). More interestingly, in addition to the same mechanism of action, TPM was found to be neuroprotective in rodent models of focal cerebral ischemia (46, 47) and experimental status epilepticus (48), which may compensate for this shortcoming of sodium valproate. Given this, we assumed that valproate and TPM might synergistically play an inhibition effect on the glutamate-related excitatory neurotransmission, which contributes to seizures decrease. However, the combination of lamotrigine (LTG) and valproate has been suggested to be particularly efficacious for epilepsy due to the synergistic effect in the previous studies (49, 50). LTG, a novel broad-spectrum antiepileptic agent, has the mechanism of action such as inhibiting the release of the excitatory amino acid glutamate through sodium channel blockade similar to TPM or valproate (51). Previous study has also demonstrated that the combination of LTG and valproate produced a supralinear “synergistic” effect based on the antiglutaminergic effect of LTG (52). Similarly, we speculated that the combination of TPM and valproate might have a similar synergistic interaction that of LTG combined with valproate. Certainly, further studies should definitely explore whether the combination of valproate and TPM is associated with additive or synergistic efficacy, and also their mechanisms of action. Comfortingly, our findings demonstrated that sodium valproate combined with TPM therapy may be a promising treatment strategy for refractory epilepsy clinically.

Our research maintains some validity. First, the included studies in our meta-analysis statistical strictly met the selection criteria. Second, no publication bias was shown, indicating that there was no bias among the pooled results. Even so, deficiencies should be taken into account when interpreting the research results. There may be several limitations to the results of this meta-analysis. First, most of the included RCTs had no descriptions of the details including blind method and allocation concealment, which may lead to deviations in implementation and measurement. Second, the published studies' quantity in our meta-analysis was insufficient, and small-sample studies may not have sufficient statistical power for estimating the relevance. Third, the current clinical data were primarily from China, with a lack of population from other countries. Finally, stratified seclusion was not described in all studies, and the corresponding risk was judged as high risk. Thus, we should carry out multicenter, randomized, strictly designed, large-scale, and double-blind research, collecting international clinical research data, so as to better assess the efficacy of sodium valproate combined with TPM vs. sodium valproate alone for refractory epilepsy. In future researches, we require higher-quality evaluations to validate our findings.

In summary, results of our meta-analysis indicated that the efficacy of sodium valproate combined with TPM was better than that of sodium valproate alone for epilepsy. This meta-analysis provides feasibility data for a larger-scale study on AED therapy of refractory epilepsy and may contribute to better therapy strategies for epilepsy clinically.

## Data Availability Statement

The original contributions presented in the study are included in the article/supplementary files, further inquiries can be directed to the corresponding author/s.

## Author Contributions

Z-YJ conceived conceptualization, performed data analysis, and supervised the project. Z-YJ and Y-QH contributed to methodology. Y-QH provided the resources and contributed to project administration. Y-QH and W-ZH performed data curation. W-ZH wrote original draft and involved in writing, reviewing, and editing. All authors contributed to the article and approved the submitted version.

## Funding

This work was funded by the Dengfeng Project for the construction of high-level hospitals in Guangdong Province, the First Affiliated Hospital of Shantou University Medical College Supporting Funding, and Publicity of the 2021 Guangdong Science and Technology Special Fund (major special project + Task list) project of Shantou Science and Technology Bureau (2021010206).

## Conflict of Interest

The authors declare that the research was conducted in the absence of any commercial or financial relationships that could be construed as a potential conflict of interest.

## Publisher's Note

All claims expressed in this article are solely those of the authors and do not necessarily represent those of their affiliated organizations, or those of the publisher, the editors and the reviewers. Any product that may be evaluated in this article, or claim that may be made by its manufacturer, is not guaranteed or endorsed by the publisher.
